# Dermal Fillers: Tips to Achieve Successful Outcomes

**DOI:** 10.4103/0974-2077.44161

**Published:** 2008

**Authors:** Maya Vedamurthy, Amar Vedamurthy

**Affiliations:** *Department of Dermatology, Apollo Hospitals and Malar Hospitals, Chennai, India*

**Keywords:** Defects, fillers, rejuvenation

## Abstract

Fillers have become a common aesthetic treatment for several cosmetic problems. Several types of fillers are available from different sources and of different longevities. It is important that the treating physician be aware of the different techniques of administration and their possible side effects. This article reviews the available literature on the subject.

## INTRODUCTION

Dermal fillers have revolutionized the field of cosmetic dermatology. With the aging of baby boomers, dermal fillers have become a sought-after rejuvenation procedure as they offer a youthful, three-dimensional look with minimal downtime.[1] Dermal fillers are gaining popularity because of the increased attention by the media and the availability of a wide array of filler materials available at prices more affordable than before. The use of fillers for soft tissue augmentation with the synergistic use of botulinum toxin and a variety of complementary procedures has become the mantra for rejuvenation.

Presently, the fillers available in India vary in source, longevity, site of deposition, and cost. The focus of this article is to analyse the fillers available in the market and to provide practical tips that would enable the dermatologist derive optimal results for the patient.

## CLASSIFICATION OF FILLERS

Fillers can be classified either on the basis of longevity in tissues, or based on their source.

### Based on longevity

Fillers are classified as temporary, semipermanent, or permanent. Temporary fillers stay in the tissue for less than a year, semipermanent fillers for up to 1–2 years, whereas permanent fillers are substances that remain in the tissue more than two years. However, there is some confusion in the classification of semipermanent and permanent status of some fillers, with some sources classifying any filler that lasts more than one year as permanent.

### Based on the origin

Fillers may be classified depending on their source as:

1. Human
2. Animal
3. Synthetic

Knowing the source of the filler is important as it helps the dermatologist to take decisions about the need for preskin testing and to know the patient’s preference. Many patients may not wish to use animal products for religious or personal reasons.

### Temporary fillers

Temporary fillers are also referred to as nonpermanent fillers. Their stay in the tissue is for less than 12 months and hence, they have the advantage of spontaneously disappearing if the patient is not satisfied with the results. Similarly, an adverse event with such fillers is also a temporary one, as seen in the majority of the cases. However, rare permanent side effects have been reported even with temporary fillers.

Some of the common temporary fillers available in India are described here:

### Bovine collagen-based products

- Zyderm 1
- Zyderm 2
- Zyplast

### Human tissue-derived collagen

- Cosmoderm
- Cosmoplast

### Synthetic fillers: Hyaluronic acid-based fillers

A wide range of hyaluronic acid products is available with varying hyaluronic acid concentrations and cross-linking structure and hence, also in their indications [Table 1].

**Table 1:** Temporary fillers, their indications, and sites of placement

Product	Description	Indication	Site of placement
Restylane	20 mg/mL	Wrinkles	Mid-dermis
SubQ	20 mg/mL	Volume filler	Subcutis
Touch	20 mg/mL	Fire lines	Superficial dermis
Vital	20 mg/mL	Hydration	Indradermal
Lipps	20 mg/mL	Lip enhancement	Lips
Perlane	20 mg/mL	Deep folds	Deep dermis
Esthelis			
Soft	20 mg/mL	Fire wrinkles and lines	Superficial dermis
Basic	22.5 mg/mL	Medium to deep wrinkle volume enhancement	Superficial to mid-dermis
Fortelis extra	25.5 mg/mL	Folds and deep wrinkles	Deep dermis
IAL system		Skin rejuvenation	Dermis
Revanesse	25 mg/mL	Wrinkles	Mid-dermis

### Semipermanent fillers

Semipermanent fillers [Table 2] undergo slow degradation with time over a period of 1–2 years. Side effects with semipermanent fillers are more common and longer lasting than temporary fillers.

**Table 2:** Semipermanent fillers, their indications, and sites of placement

Products	Description	Indication	Site of placement
Sculptra	Poly-L-Lactic acid	Volume filler	Deep dermis, subcutis
Radiesse	Calcium hydroxylapatite	Volume enhancement	Deep dermis
Bioin blue	Polyvinyl alcohol	Volume enhancement	Hypodermis Deep dermis intramuscular subfascial

### Permanent fillers

Permanent fillers are fillers that remain for longer than two years in the tissyue. Side effects with permanent fillers tend to be more permanent, and complication is the main issue with any permanent filler. Commonly used permanent fillers available are shown in Table 3.

**Table 3:** Permanent fillers, their indications, and sites of placement

Products	Description	Indication	Site of placement
Aquamid	Polyacrylamide	Deep folds, wrinkle volume correction	Subcutis
Amazingel	Polyacrylamide	Deep folds, wrinkle volume correction	Subcutis
BioAlcamid	Polyalkylamide	Volume enhancement for large defects	Subcutis

Other materials, which are less commonly used, include Artefill and Silicone.

## INDICATIONS OF DERMAL FILLERS

Fillers are mainly used in the rejuvenation of facial areas. However, dermal fillers may also be used in nonfacial areas and for cutaneous defects.

### Facial areas

- Wrinkles and folds
- Lip Augmentation
- Depressed scars – postsurgical, traumatic, postacne, chickenpox, and other diseases
- Enhancement of facial contour
- Periocular melanoses and sunken eyes
- Dermatological diseases – angular cheilitis, dermal atrophy, AIDS lipodystrophy
- Earring ptosis, atrophic earlobes
- Nasal depressions

### Nonfacial areas

- Neck
- Décolleté    }        Rejuvenation
- Hands
- Corns and calluses – to reduce contact points, pain, and risk of ulcer formation[2]

## CONTRAINDICATIONS

Complications of fillers are generally rare; they include:[3]

### Absolute

- Hypersensitivity to products
- Unrealistic expectations

### Relative

- Keloidal tendency
- Patients with autoimmune disease

## PATIENT EDUCATION AND ASSESSMENT

Patients should be counseled about the nature of the filler, what to expect, longevity, any possible side effects, and the cost.[4] An assessment of both the physical and psychological states of the patient is necessary for a successful outcome. Written informed consent ensures compliance with regulatory and legal requirements as well as the management of the patient’s expectations. Pre- and post- photographs are important to critically analyse the results of the treatment and also, to prepare for medico-legal situations.

## PROCEDURE OF FILLER INJECTION

The aesthetic benefit for the patient with temporary fillers can be attributed to 90% technique and 10% substance, whereas it is usually 99% technique with permanent fillers.[3]

### Preparation and anaesthesia

The area of injection and also the surrounding skin should be cleaned properly with antiseptics. Anaesthesia is important for technical benefit and the patient’s comfort. Anaesthesia can be ensured by:

- Application of ice
- Topical EMLA cream application
- Regional nerve blocks – infraorbital, mental, maxillary, submucosal – as applicable, depending on the area
- Distraction Techniques such as massage, application of vibration
- Talking in a soothing and comforting manner – Talkesthesia

### Injection technique

The choice of the injection technique depends on the indication, its location, the filler substance, size of the needle, and the experience of the injector. The techniques include:[5]

1. Linear threading technique
2. Serial puncture
3. Fanning
4. Cross-hatching
5. Depot
6. Fern
7. Cone

The first four techniques are used commonly, whereas the last three are only used in special situations. It is important to place the filler in the right place and the bevel orientation is not a significant issue at any site.

### Postinjection management

Patients should be asked to avoid extreme cold or heat for 48 hours. Massaging of the treated area and strenuous physical activity should be avoided for six hours. Patients are asked to sleep with their heads elevated for one night; skin care routine may be followed after 24 hours.

### Measures to achieve successful outcomes

A comprehensive treatment plan should be devised to suit the needs of each patient. Choosing the right filler for the right indication is vital. Where essential, combinations of fillers with botulinum toxin should be used to optimize the results.[6] In such cases, it is advisable to inject the botulinum toxin first, wait for a week to see the improvement, and then, inject the filler to achieve best results.

## FACIAL ANATOMIC AREAS: SPECIAL CONSIDERATIONS

### Glabella area

Always choose patients with a positive glabella test. Inject small boluses relatively superficially, using only moderate pressure while injecting. Always watch for erythema and stop injecting when you see blanching as this will reduce the incidence of ischaemic necrosis which is common in this area. Techniques used in this area are usually a combination of linear threading and serial puncture techniques.

### Nasolabial fold

This is the ideal indication to start with for a novice injector. Stretch and compress the skin to visualize the fold. Inject into a site medial to the fold to avoid further cheek ptosis. Overcorrection is common here and this leads to change in animation, lumpiness, and bumpiness. Remember that nasolabial folds always display a degree of natural asymmetry which should be discussed with the patient prior to injection. Usually a linear threading or a fern technique is chosen to treat this area.

### Lips

The lips are the focal centre of the lower face. It is always important to consider antiviral prophylaxis in a patient with a history of herpes simplex episodes.

The height of the lips and the proportion of the upper to lower lip should be considered. The lower lip should protrude slightly beyond the upper lip. The upper lip should be 2/3 of the volume of the lower lip. Usually lip augmentation is done with a larger particle-sized filler in contrast to lip line augmentation which is done with a smaller gel particle-sized filler. Overcorrection of the upper lip can lead to a “duck bill” appearance. As lips swell immediately following the injection of the filler, steroid prophylaxis needs to be considered.[7]

A linear threading technique is done for volume augmentation and a microdroplet technique is used very rarely when one has to inject near the submucosal area.

### Periocular area

The best candidates are younger patients with thicker skin and minimal to moderate volume loss. A depot injection at the level of the periosteum essentially traps the gel implant creating a long-lasting effect. Edema and ecchymosis are quite common in this area. Too superficial placement or thin skin in this area can lead to visibly pale nodules or Tyndall effect. Injection of filler into the periocular area is a difficult procedure and only a person with good training and experience should attempt it. One should also be aware of serious side effects such as blindness (see under complications).

## COMPLICATIONS OF FILLERS

Temporary fillers have fewer complications and transient adverse effects when compared to semipermanent and permanent fillers. Adverse effects could be injection-related or filler material-related and may appear either immediately or later.[7]

### Early complications

- Hypersensitivity reactions
- Haematomas and ecchymoses
- Infections – reactivation of herpes simplex
- Nonhypersensitivity-related swelling
- Acneiform eruptions
- Erythema – transient or permanent
- Skin necrosis
- Embolism (blindness)
- Tyndall effect

### Late complications

- Implant migration
- Telangiectasia
- Granulomas
- Lipoatrophy
- Hypertrophic scarring
- Sterile abscess

The treatment of complications should be aggressive and initiated as soon as possible after occurrence. Effective treatment of complications and assuring patients in the interim are the keys to successful practice.

## NOVEL THERAPEUTIC AGENTS AS DERMAL FILLERS

### Stem cells

Adipose tissue is easily obtained through liposuction and it provides a good source of stem cells which serve as a useful agent for soft tissue regeneration or repair. It has been found that fatty tissue has the highest percentage of adult stem cells of any tissue in the body with about 5000 adipose-derived stem cells per gram of fat *vs* 100–1000 stem cells per milliliter of bone marrow.

### Autologous vein transplantation

Autologous vein transplantation[9] can be performed with the unwanted veins of the patient. The procedure involves extracting varicose or other unwanted leg or hand veins of the patient, soaking the vein in hypertonic saline to destroy endothelial cells, and then, implanting portions of the remaining material (containing collagen, elastin, and muscle) into the dermal defect. The endothelium is destroyed to prevent newer angiogenesis in the implanted area. So far the results have been long-lasting with no complications. Although results are encouraging, this procedure is laborious at present, and is restricted to patients with unwanted veins.[8]

## CONCLUSION

With a variety of fillers flooding the market and with the aging of baby boomers aspiring to retain their youthfulness, careful selection of the patient, the dermal filler, and the technique can maximise patient satisfaction and lead to a successful outcome

## References

[1] Klein, Elson (2000). The history of substances for soft tissue augmentation. Dermatol Surg.

[2] Matarasso SL, Carruthers JD, Jewell ML (2006). Restylane Consensus Group. Restylane for soft tissue augmentation. Plast Reconstr Surg.

[3] Lemperle G, Rullan PP, Gauthier-Hazan N (2006). Dermal filler complications. Plast Reconstr Surg.

[4] Vedamurty M (2004). Soft tisssue augmentation. Indian J Dermatol Venreol Leprol.

[5] Klein AW, Nouri Kevyan, Leal Khouri Susana (2003). Temporary Fillers. Techniques in Dermatologic Surgery.

[6] Naoum C, Plakida DD (2001). Dermal filler materials and botulin toxin. Int J Dermatol.

[7] Boulle KD (2004). Fillers’ complications. J Cosmet Dermatol.

[8] Moseley TA, Zhu M, Hedrick MH (2006). Adipose-derived stem and progenitor cells as fillers. Plast Reconstr Surg.

[9] Goldman MP (2007). Optimizing the use of fillers for facial rejuvenation: The right tool for the right job. Cosmet Dermatol.

